# ADHD, stress, and anxiety

**DOI:** 10.3389/fpsyt.2025.1536207

**Published:** 2025-03-18

**Authors:** Petr Bob, Michal Privara

**Affiliations:** Center for Neuropsychiatric Research of Traumatic Stress, Department of Psychiatry and UHSL, First Faculty of Medicine, Charles University, Prague, Czechia

**Keywords:** ADHD, anxiety, depression, stress, developmental disintegration, primitive reflexes, neural interference

## Abstract

Recent findings on stress and anxiety in attention deficit hyperactivity disorder (ADHD) suggest that specific processes related to brain developmental disorganization could create a vulnerable background that increases sensitivity to stress stimuli from the psychosocial environment. These basic neurodevelopmental processes are closely related to the developmental mechanisms of primitive functions and their integration or disintegration. In this context, the psychopathological processes that manifest in ADHD are linked to the mechanisms of disturbed inhibitory functions that may cause incongruent neural interactions (“neural interference”) between the more primitive functions and the higher levels of attentional and cognitive neural processes. These disturbed developmental processes may also determine increased sensitivity to stressful experiences that, in ADHD cases, could lead to the manifestations of various psychopathological symptoms such as disturbed attentional and motor functions, anxiety, and depression, among other cognitive and affective disturbances. These findings, based on previous research, suggest novel framework and hypothesis on how this neurodevelopment-based increased sensitivity to stress stimuli could manifest in the etiopathogenesis of ADHD in its relationship with cognitive, affective, and motor deficits.

## Introduction

Recent findings related to research on the retained primitive reflexes in patients with attention deficit hyperactivity disorder (ADHD) indicate that the basic processes in ADHD etiopathogenesis may be related to dysfunctions in hierarchical organization during central nervous system (CNS) development when the different brain developmental stages are interconnected on various hierarchical and functional levels (1, 2). Uncovering these processes in more detail is extremely important for future treatment strategies and for the understanding of the complex etiopathogenesis of the disease. Current findings suggest that, in cases of dysfunctional neural development, these hierarchical and functional levels may manifest incongruent interactions (the so-called neural interference) between the early and the later developed brain functions during ontogenesis (1, 3). This neural interference may manifest in the case when the emergence of a new function that should have inserted the older one is not related to the diminishing or sufficient inhibition of this older function, which could lead to “neural disintegration” caused by incongruent neural processes. In this context, recent findings focused on the etiopathogenesis of ADHD suggest that these processes related to brain developmental disorganization could create a vulnerable background that increases sensitivity to stress stimuli from the psychosocial environment (2, 4). This increased sensitivity to stress stimuli that might occur in the etiopathogenesis of ADHD could then be related to various forms of cognitive, affective, and motor deficits that often manifest in individuals with ADHD (1, 3, 4). In this biopsychosocial context, this conceptual analysis focused on recent findings on the retained primitive reflexes in ADHD (2, 3) and provides novel perspectives into understanding the multiple etiopathogenetic factors of this condition. The main focus and objectives of the analysis are the interactions of the neurobiological developmental mechanisms with stress influences in the etiopathogenesis of ADHD, with main implications for developmental disorganization in its relationship with cognitive and affective disintegration and the related psychopathological symptoms that could manifest in ADHD. The currently available models and theories on the pathogenesis of ADHD are mainly focused on the neurobiological developmental mechanisms of this condition, but do not explain how these neural processes during development might interact with stress influences from the psychosocial environment. With this aim to link the neurobiological findings on the pathogenesis of ADHD, we briefly summarize the diagnostic definitions of ADHD to show how basic conceptualizations of “organic” minimal brain dysfunctions change during the time for a more complex understanding of this condition. From this historical perspective, this requirement for a more detailed understanding was mainly influenced by findings that the psychosocial environment and mainly stress stimuli strongly interact with the developmental abnormalities specifically related to the etiopathogenesis of ADHD.

## Definitions and epidemiology of ADHD

ADHD represents a historically heterogeneous concept that started with the introduction of “minimal brain dysfunction” by Still in 1902 (5), who provided detailed descriptions of the hyperactivity and hyperkinetic symptoms. Much later, in the 1970s, attentional dysfunctions were described by Douglas (6). The historical term “minimal brain dysfunction” was replaced in 1968 by the conceptualization and definition of hyperactivity, but even then was still understood mainly as a result of some biological origin more than of environmental causes (7–9). This concept was later incorporated into the official diagnostic nomenclature described in the second edition of the Diagnostic and Statistical Manual of Mental Disorders (DSM-II) (7, 10) as a disorder characterized by overactivity, restlessness, distractibility, and short attention span, particularly in young children, that usually diminishes in adolescence (7–9, 11).

The diagnostic definitions of ADHD in the last decades have reflected an increased knowledge of this condition and its developmental etiopathogenesis. Its recent definitions included in basic diagnostic classification systems, such as the International Classification of Diseases, 10th revision (ICD-10), DSM-IV, and DSM-V, describe the behavioral characteristics of ADHD as related to deficits in “executive functions” that negatively influence the control and regulation of cognitive processes and “self-control” (12). In the case of ADHD as a typical developmental disorder, these neurocognitive characteristics often manifest in the different ontogenetic stages from early childhood to adulthood, which are mainly related to specific deficits in attentional and executive functions (13, 14).

Most typical symptoms according to DSM-IV-TR (DSM-IV, text revision) include excessive motor activity, inattention, and impulsiveness that manifest in childhood (11), while according to the DSM-V definition (15), ADHD is characterized by a pattern of behavior that can be divided into two categories: inattention, and hyperactivity and impulsivity. Children must have at least six symptoms from either (or both) the inattention group of criteria and the hyperactivity and impulsivity criteria, while older adolescents and adults (over 17 years old) must show at least five symptoms. According to DSM-V, symptoms of ADHD must be present prior to age 12 years, compared to 7 years as the age of onset in DSM-IV. In addition, DSM-V does not include the exclusion criteria for people with autism spectrum disorder as the symptoms of both disorders may co-occur.

According to epidemiological data, symptoms of ADHD developed later in adolescence are very similar to ADHD in children with typically increased attentional deficits that are more frequent than hyperactivity and impulsivity, and the treatment procedures in adolescence are also very similar (2, 16–18). Reported evidence also shows that adult symptoms of ADHD, due to disturbances of the executive functions, are significantly related to a higher prevalence of antisocial personality and behavioral disorders. These data show that antisocial personality disorder manifests in 12%–28% of adults with ADHD (in healthy controls, it is 2%–8%), while behavioral disorders manifest in 22%–62% of adults with ADHD (only 4%–8% in healthy controls) (2, 14, 17–25). These highly prevalent antisocial personality and behavioral disorders in adults with ADHD are also related to increased manifestations of criminal behavior, mood disorders, anxiety disorders, and addictive behavior in comparison to healthy controls (19, 25–27). In addition, reported data have shown that antisocial behavior in children is often related to the same difficulties in adulthood in approximately 20%–45% of adults with an ADHD diagnosis (2, 7, 14, 18, 20). For example, according to reported data, approximately 10% or more of individuals in prison have a diagnosis of ADHD (28, 29).

Important prediction factors of later difficulties and bad prognosis are also early manifestations of symptoms of ADHD, and reported data have shown that later manifestations of these ADHD symptoms indicate better prognosis (30–33). Other very important negative factors for future prognosis represent the early occurrence of oppositional defiant disorder, mood disorder and anxiety, and a lower level of intelligence (2, 17, 19, 25, 34, 35). Major negative factors also represent the occurrence of psychopathology in parents, ADHD in other family members, the social and economic status of the family, and frequent conflicting situations and psychosocial stress (2, 24, 25, 34, 36).

## Executive functions, psychosocial stress, and ADHD

The major results in recent ADHD research show that the processes of executive control are typically affected in ADHD and mainly include dysfunctions in inhibitory control (2, 37–39). In addition, recent empirical findings and theoretical conceptualizations indicate that, together with inhibitory dysfunctions, increased emotional excitation may play a role in ADHD deficits. For example, the “cool” cognitive deficits in executive functions are closely linked to attentional dysfunctions; on the other hand, “hot” deficits are related to the dysfunctional ability to process emotional information that produces hyperactivity and impulsivity (2, 39–42). These recent findings indicate that ADHD cannot be explained solely as a consequence of frontal lobe executive dysfunctions. An important role can also be attributed to emotional dysregulation associated with increased excitability in the limbic system, which may cause ADHD disturbances even when frontal executive dysfunctions are not a primary factor in the etiopathology of ADHD (39–42).

Altogether, these findings suggest the so-called dual-pathway concept of the two basic developmental trajectories that could lead to ADHD (43). The first is represented by frontal executive dysfunctions (2, 39, 42–44), while the second is mainly linked to dysfunctions in brain systems related to emotions and motivation (45).

Recent findings suggest that attentional and executive dysfunctions may be related to impulsivity, often observed in ADHD, which can also contribute to social dysfunctions and increased vulnerability to stress-related influence (39). For example, recent findings show that children with ADHD often manifest antisocial behavior, most likely due to deficits in executive functions, impulsivity, and aggressive behavior related to stressful situations (4, 39, 42). Impulsivity is often associated with antisocial behavior, which occurs in 20%–45% of adults with ADHD and contributes to interpersonal problems (14, 18, 20). According to some data, approximately 10% or more of individuals from populations who display various forms of criminal behavior have ADHD (2, 14, 28, 29).

According to epidemiological data, ADHD is related to significantly increased levels of mental stress (4, 39, 46, 47). For a more detailed understanding of how stress could influence individuals with ADHD, it seems extremely important to note that the various functional changes in ADHD and posttraumatic stress disorder (PTSD) are frequently similar, and it is possible to expect that the different processes described in stress-related disorders are extremely important in the etiopathogenesis of ADHD (4, 36, 39, 48, 49).

In this context, recent evidence indicates that experiencing traumatic events or repeated stressors in childhood often may cause severe mental problems that could have delayed effects and lead to various neurobiological changes that influence attentional dysfunctions, disturbed cognitive control, and emotional dysregulation (4, 39, 50–52). The development of ADHD is also linked to deficits of neural mechanisms that might underlie specific changes in attentional functions and decreased cognitive control, often associated with impaired inhibitory functions (2, 39–44).

## Brain developmental stages, neural disintegration, and ADHD

According to neurodevelopmental findings, later developed functions during the ontogenesis of the CNS tend to replace the older ones when higher stages of CNS development have been successfully achieved (1, 53, 54). The development of neural functions based on ontogenetically successive complex neuronal levels enables the performance of more adaptive functions; on the other hand, disinhibition or the release of developmentally older functions from inhibitory control manifests in various neurological and psychiatric disorders (1, 2, 54).

As recent findings show, the highest risk of neural disintegration is during the sensitive developmental stages of brain functions that are also particularly vulnerable to various insults, such as brain damage, toxic influences, or psychological stress (4, 55–57). The particularly important postnatal developmental deficits of higher motor and cognitive functions that likely also have various etiological backgrounds are persisting “primitive reflexes” (3, 58–60), such as symmetric tonic neck reflex (STNR) and asymmetric tonic neck reflex (ATNR), among others (59, 61). These primitive (or primary) reflexes (3, 62, 63) present specific forms of innate “behavioral movement patterns” (64) that are replaced by higher motor and cognitive functions (58–60), and when they occur in the later stages of development, they may present a form of “soft neurological signs” (65).

In this context, recent clinical evidence indicates that manifestations of primitive reflexes in later age than is ontogenetically typical are likely linked to frontal lobe dysfunctions and cortical disinhibition and may occur in various neuropsychiatric syndromes such as ADHD, schizophrenia, depressive and anxiety disorders (3, 66), dementia and Parkinsonism (67), and delirium (68), among other neuropsychiatric disorders (59, 60, 69). These data suggest that persistent (or retained) primary reflexes in general represent evolutionary lower levels of neurophysiological processes that may interfere with processing on higher levels and cause neural disintegration, which may be linked to different neuropsychiatric conditions including ADHD, anxiety, mood disorders, and other mental disorders (1, 54).

## Stress, ADHD, and anxiety

According to current evidence, ADHD shares a high comorbidity with anxiety disorders. These findings show that symptoms of anxiety may increase the symptoms of ADHD; on the other hand, deficits of executive functions related to ADHD may increase anxiety (25, 70). Nevertheless, the causal relations where the anxiety would predict ADHD, or the ADHD would predict anxiety, were not found, indicating that both diagnoses could coexist as comorbidities. However, there is no evidence that ADHD could create anxiety as its symptom or that anxiety would implicate ADHD (25, 71). These findings are in agreement with the “dual-pathway” trajectory in the etiopathogenesis of ADHD based on the two interacting systems, where the first is represented by frontal executive dysfunctions and the second is mainly linked to dysfunctions in brain systems related to emotions and motivation (39, 42). This mutual comorbidity and “interplay” between ADHD symptoms and anxiety indicates that inhibitory deficits specifically interact with emotional excitation related to stress stimuli, and the dysfunctional inhibitory systems could cause higher vulnerability with respect to stress stimuli from the social environment (2, 4). On the other hand, in cases of ADHD where the executive dysfunctions are not the main etiopathogenetic factors, increased emotional excitation caused by stress stimuli may also play a role in ADHD deficits and symptoms (2, 4). This interplay between executive dysfunctions and emotional dysregulation due to stressful experiences may then also implicate the observed comorbidities and relationships between the attentional symptoms related to ADHD and the anxiety-related emotional dysregulation in patients with ADHD (2, 4). This interplay between the attentional symptoms of ADHD and the symptoms of anxiety also reflects the dual pathway between the “cool” cognitive deficits mainly related to executive dysfunctions and the “hot” deficits related to the dysfunctional ability to process emotional information that often may produce anxiety, hyperactivity, and impulsivity (39–42). These recent findings on the relationship between ADHD symptoms and anxiety also confirm that ADHD and its symptoms cannot be explained only as a consequence of frontal lobe executive dysfunctions and that important influences on the etiopathogenesis of ADHD are also related to emotional dysregulation that is closely linked to increased excitability in the limbic system (4, 39, 42).

## Conclusion

Recent findings suggest that the etiopathogenesis of ADHD could represent a process related to the “incongruent interactions” of the more primitive neural mechanisms, such as the primitive reflexes with higher levels of brain functions, due to an insufficiently developed cognitive and motor integration. This developmental disintegration is also related to the disturbed balance in ADHD (2, 3). In some cases of ADHD, these retained reflexes and incoordination are related to the disturbed balance and attentional dysregulation linked to incongruent interactions (or conflict) between the higher and lower levels of cognitive and motor functions during brain processing (46, 47).

Recent findings also show that a high proportion of individuals with ADHD manifests altered balance and motor abnormalities (72–74). According to brain imaging studies, these balance deficits are likely linked to prefrontal cortex deficits that influence the attention and executive functions (75–77). These dysfunctions could also have a cerebellar origin: individuals with ADHD, in many cases, exhibit atrophy in the cerebellar regions associated with balance and gait control, and these balance and motor dysfunctions are linked to inhibitory deficits due to cerebellar abnormalities (72, 78–80).

In future research, this relationship between the dysregulation of emotional systems and executive dysfunctions could also help in understanding the unresolved relationship between internalizing the symptoms of ADHD, which are mainly related to anxiety and depression, and externalizing the symptoms related to behavioral dysfunctions, which mainly manifest as conduct problems, aggressive behavior, and oppositional defiant disorder (2).
